# EMANCIPATORY PEDAGOGY IN GERONTOLOGICAL SPACES

**DOI:** 10.1093/geroni/igad104.2025

**Published:** 2023-12-21

**Authors:** Brittney Pond, Nicholas DiCarlo, Brittney Pond

## Abstract

Awareness of the field of gerontology has been bolstered by increased training and education programs and other critical pedagogies in several disciplines in higher education. Public and philanthropic initiatives have also supported the development of scholars and researchers in this field (Gross & Eshbaugh, 2011; Merz et al., 2017; Snyder, Wesley, Lin & May, 2008; Wesley, 2005). Furthermore, research notes that incorporating experiences of LGBTQ+ older adults in gerontological spaces can help to address a lack of representation in higher education and provide critical training to support LGBTQ+ older adults (Lipinski, Wilson, Kortes-Miller & Stinchcombe, 2022; Smith, Altman, Meeks & Hinrichs, 2019). This symposium aims to discuss critical pedagogies and emancipatory gerontology in higher education through an intersectional lens. We aim to address the question, where do critical pedagogies flourish, where are they needed, and in what ways? Presenters discuss multiple aspects of this question, describing the ways in which access is a critical component of emancipatory pedagogies, including 1) how we learn and teach abolitionist gerontology in interdisciplinary spaces 2) confronting ableism and ageism in gerontological spaces as part of intergenerational pedagogical praxis and 3) highlighting the need for LGBTQ+ aging education in curricula by discussing a student-led “queering gerontology” campaign with tangible outcomes.

